# The role of flow cytometry in the classification of myeloid disorders

**DOI:** 10.1007/s00292-023-01272-8

**Published:** 2023-11-22

**Authors:** Leonie Saft

**Affiliations:** Clinical Pathology and Cancer Diagnostics, Karolinska University Hospital and Institute, 171 76 Stockholm, Sweden

**Keywords:** Flow cytometry, Myeloid neoplasms, Classification, Immunophenotyping, Artificial intelligence, Durchflusszytometrie, Myeloische Neoplasien, Klassifikation, Immunphänotypisierung, Künstliche Intelligenz

## Abstract

The World Health Organization classification (WHO-HAEM5) and the International Consensus Classification (ICC 2022) of myeloid neoplasms are based on the integration of clinical, morphologic, immunophenotypic, and genomic data. Flow cytometric immunophenotyping (FCIP) allows the identification, enumeration, and characterization of hematopoietic cells, and is therefore a powerful tool in the diagnosis, classification, and monitoring of hematological neoplasms. The vast majority of flow cytometry (FCM) studies in chronic myeloid neoplasms focus on its role in myelodysplastic neoplasms (MDS). FCM can also be helpful for the assessment of myeloproliferative neoplasms (MPN) and MDS/MPN, including the early detection of evolving myeloid or lymphoid blast crisis and the characterization of monocytic subsets. The classification of acute myeloid leukemia (AML) is primarily based on cytogenetic and molecular findings; however, FCIP is needed for subclassification of AML, not otherwise specified (NOS; ICC)/AML defined by differentiation (WHO-HAEM5). The main role of FCM in AML remains in making a rapid diagnosis and as a tool for measurable residual disease monitoring. Machine learning and artificial intelligence approaches can be used to analyze and classify FCM data. This article, based on an invited lecture at the 106th Annual Meeting of the German Society of Pathology in 2023, reviews the role of FCM in the classification of myeloid neoplasms, including recent publications on the application of artificial intelligence.

## Abstract (de)

Die Klassifikation gemäß World Health Organization (WHO-HAEM5) und die International Consensus Classification (ICC) myeloischer Neoplasien basieren auf der Integration klinischer, morphologischer, immunphänotypischer und genetischer Daten. Die durchflusszytometrische Immunphänotypisierung (FCIP) ermöglicht die Identifizierung, Auszählung und Charakterisierung von hämatopoetischen Zellen und ist daher ein leistungsstarkes Instrument bei der Diagnostik, Klassifikation und Verlaufskontrolle hämatologischer Neoplasien. Die Mehrzahl der Studien über die Anwendung der Durchflusszytometrie (FCM) bei chronischen myeloischen Neoplasien fokussieren auf die Rolle bei der Diagnostik myelodysplastischer Syndrome (MDS). FCM ist auch hilfreich bei der Beurteilung myeloproliferativer Neoplasien (MPN) und von MDS/MPN, einschließlich des frühen Nachweises einer drohenden myeloischen oder lymphatischen Blastenkrise und der immunphänotypischen Charakterisierung monozytärer Subpopulationen. Die Klassifizierung der akuten myeloischen Leukämie (AML) beruht primär auf zytogenetischen und molekularen Befunden; FCIP ist jedoch erforderlich für die Subklassifikation der AML, die nicht anderweitig spezifiziert ist (NOS; ICC)/AML, die definiert ist durch Differenzierung (WHO-HAEM5). Die wichtigste Rolle der FCM bei AML ist die schnelle Diagnostik und die Anwendung zur Überwachung messbarer Resterkrankung. Maschinelles Lernen und künstliche Intelligenz können für die Analyse und Klassifizierung von FCM-Daten genutzt werden. Dieser Artikel, der auf einem Vortrag bei der 106. Jahrestagung der Deutschen Gesellschaft für Pathologie 2023 basiert, ist eine Zusammenfassung zur Rolle der Durchflusszytometrie bei der Klassifikation myeloischer Neoplasien, einschließlich neuerer Publikationen zur Anwendung von künstlicher Intelligenz.

Flow cytometry is an indispensable tool in the diagnosis and monitoring of hematologic neoplasms. It allows the identification, enumeration, and characterization of hematopoietic cells in the peripheral blood and bone marrow. Immunophenotypic characteristics are included as diagnostic criteria in the fifth edition of the WHO classification (WHO-HAEM5) and the International Consensus Classification (ICC). This article reviews the role of flow cytometry in the classification of myeloid neoplasms, including recent publications on the application of artificial intelligence.

Flow cytometric immunophenotyping (FCIP) is a powerful laboratory tool for diagnosis, classification, and monitoring of hematologic neoplasms. In the past decades, clinical flow cytometry (FCM) has evolved from a technique primarily used to characterize large populations of abnormal cells to one that can also routinely evaluate small cell populations for subtle aberrancies in antigen expression. These advances have expanded and refined the clinical applications of FCM. The WHO-HAEM5 [14] and the ICC [1] endorse a multiparametric, integrated approach to diagnosis and outline the morphologic, immunophenotypic, and genetic features characteristic of each disease entity.

The detailed characterization of immunophenotypic alterations in chronic myeloid neoplasms, while not entity specific, can lend support to a neoplastic diagnosis and assist in the differential diagnosis. In the past, FCIP approaches have focused on the analysis of myeloid progenitors (blasts), since these are invariably affected irrespective of disease characteristics (acute vs. chronic) and are likely more stable than maturing myelomonocytic cells. However, immunophenotypic alterations are also frequently present in the maturing myelomonocytic compartment, particularly in myelodysplastic neoplasms (MDS), and partitioning of monocytic subsets is now used as a co-criterion in chronic myelomonocytic leukemia (CMML). Therefore, there is a clear role of FCIP in routine diagnostic algorithms and for the classification of chronic myeloid neoplasms. Immunophenotypic alterations and their significance are briefly summarized in Table 1, including a literature review on the use of FCM in major categories of myeloid neoplasms.

**Table 1:** Flow cytometric immunophenotyping in the diagnosis and classification of myeloid neoplasms

Major WHO-HAEM5/ICC categories of myeloid neoplasms	Immunophenotypic characterization/alteration	Significance	Citation no.	Literature review
				reference by author/year/journal
MDS	Broad MDS panel with a core set of 17 markers according to European Leukemia Network (ELN) guidelines; MDS scores, reviewed in [19]; screening panel for cases with unclear cytopenia [22]	High concordance of aberrant findings with cytomorphology; >3% CD34^+^ myeloid progenitors highly associated with MDS	[1, 12, 14, 19–22, 28, 29]	Shameli et al. (2021), *Cytometry B Clin Cytom* 100
				Chan et al. (2023), *Cytometry B Clin Cytom* 104
				Subira et al. (2021), *Ann Hematol* 100
				Refer to Table 2 for additional references
MPN	Myeloid progenitors (quantification; aberrant immunophenotype) Alterations in maturing myelomonocytic cells; use of “monocyte assay”	Distinction between MPN with monocytosis & CMML; quantification & characterization of blasts Detection of evolving lymphoblastic crisis in CML; distinction between MPN-AP & de novo acute leukemia	[8, 18, 26]	Ouang et al. (2015), *Cytometry B Clin Cytom *88
				Bassan et al. (2022), *Med Oncol* 39
				Kern et al. (2013), *Cytometry B Clin Cytom 84*
				Mannelli et al. (2022), *Am J Hematol* 97
				Guglielmelli et al. (2017), *Blood* 129
				Jeryczynski et al. (2017), *Am J Hematol* 92
				Bardet et al. (2015), *Haematologica* 100
				El Rassi et al. (2015), *Cancer* 121
				Chan et al. (2023), *Cytometry B Clin Cytom* 104
MDS/MPN	MDS-type immunophenotypic alterations; quantification & characterization of myeloid progenitors; immunophenotypic characterization of monocytic subsets	Abnormal partitioning of monocytic subsets as diagnostic criterion in CMML (versus other myeloid neoplasms with monocytosis versus reactive monocytosis)	[26]	Patnaik et al. (2017), *Blood Cancer J* 7
				Wagner-Ballon et al. (2023), *Cytometry B Clin Cytom *104
				Solary et al. (2020), *Best Pract Res Clin Haematol* 33
				Hudson et al. (2018), *Leuk Res* 73
				Kern et al. (2013), *Cytometry B Clin Cytom* 84
				Shameli et al. (2021), *Cytometry B Clin Cytom *100
				Li et al. (2021), *Am J Clin Pathol *156
				Huang et al. (2016), *J Clin Pathol* 69
				Maioli et al. (2016), *Leuk Lymphoma* 57
				Cargo et al. (2019), *Blood* 133
M/LN-eo-TK	Broad myeloid/lymphoid FCM panels	Identification of small subpopulations, not apparent by morphology (immunohistochemistry); lineage infidelity; aberrant mast cells	[1, 14, 23, 24]	A large number of single case reports & literature reviews on specific subcategories of M/LN-eo-TK (recently reviewed in [24]) illustrate how FCM can assist in establishing the diagnosis
Mastocytosis	Aberrant immunophenotype, including expression of CD2, CD25, and/or CD30	Included as diagnostic criterion; detection of associated myeloid neoplasm or concurrent lymphoid or plasma cell neoplasm	[1, 14]	Pardanani et al. (2009), *Blood *114
				Pardanani et al. (2016), *Leukemia* 30
				Morgado et al. (2013), *Histopathology* 23
				Russano de Paiva et al. (2018), *Medicine *(Baltimore) 97
AML defining genetic abnormalities; AML defined by differentiation (WHO-HAEM5)/AML, NOS (ICC)	Phenotype–genotype correlation (recently reviewed in [17]); monocytic/megakaryocytic differentiation; minimal differentiation	Rapid diagnosis in APL; guidance for additional testing; MRD monitoring; FCIP required for AML lacking defining genetic abnormalities (update on differentiation markers in WHO-HAEM5/ICC)	[1, 3, 5, 6, 14, 17, 27]	Merati et al. (2021), *Front Oncol* 11
				Xiao et al. (2021), *Blood* 137
				Wang et al. (2022), *Cancers* 14
BPDCN	Expression of CD123 and one other pDC marker in addition to CD4 and/or CD56; or expression of any three pDC markers & absence of all expected negative markers	Immunophenotypic diagnostic criteria	[1, 14]	Wang et al. (2022), *Cancers* 14
				Khoury et al. (2018), *Curr Hematol Malignancy Reports* 13
				Deotare et al. (2016), *Am J Hematol* 91
				Wang et al. (2021), *Haematologica *106

*MDS* myelodysplastic syndrome, *MPN* myeloproliferative neoplasms, *MPN-AP* accelerated phase of MPN, *MDS/MPN* myelodysplastic/myeloproliferative neoplasms, *CMML* chronic myelomonocytic leukemia, *M/LN-eo-TK* myeloid/lymphoid neoplasm with eosinophilia and tyrosine kinase gene fusion, *AML* acute myeloid leukemia, *NOS* not otherwise specified, *APL* acute promyelocytic leukemia, *MRD* measurable residual disease, *BPDCN* blastic plasmactyoid dendritic cell neoplasm

This article, based on an invited lecture at the 106th Annual Meeting of the German Society of Pathology in 2023, is primarily focused on myelodysplastic neoplasms (MDS), myeloproliferative neoplasms (MPN), and MDS/MPN. Experiences from recent bone marrow (BM) workshops on eosinophilic disorders and mastocytosis, including myeloid/lymphoid neoplasms with eosinophilia and tyrosine kinase gene fusions (M/LN-eo-TK), provide evidence that FCM has an important role in recognizing these rare entities [23, 24]. Finally, FCIP remains important for rapid diagnosis and measurable disease monitoring of acute myeloid leukemia (AML) and is required for the subclassification of AML, not otherwise specified (NOS; ICC)/AML defined by differentiation (WHO-HAEM5). The application of artificial intelligence (AI) and machine learning (ML) in the analysis of FCM data in clinical hematology is discussed, including a literature review with focus on recent publications (Table 2).

**Table 2:** Artificial intelligence and machine learning: a literature review with focus on recent publications on the use of flow cytometry in clinical hematology

Reference	Type of disease studied/task	Type of AI tool/application	Significance
Author/year/journal			
*General review*			
Duetz et al. (2020), *Curr Opin Oncol* 32	Computational analysis of FCM data in hematological malignancies; *literature review*	Two main types of computational methods; dimensionality reduction & clustering	Increase ease of use, objectivity & accuracy of FCM data analysis; integration with digital pathology approaches
Bene et al. (2021); [2]	AI applications with focus on hematological neoplasms, including clinical examples	Various AI tools for automated, unsupervised FCM analysis; FlowSOM	Integration of FlowSOM & existing FCM software programs (e.g., Kaluza, Beckman Coulter) links unsupervised & supervised analysis
Shouval et al. (2021), Br *J Haematology* 192	AI applications in clinical hematology including *literature review*	Various references on supervised ML studies in hematology	Provides tools & guidance for understanding ML and its applications
*Bone marrow and peripheral blood*			
Lacombe et al. (2019), *HemaSphere* 3	Normal or diseased BM subsets	FlowSOM; Kaluza software program	Objective delineation of BM differentiation pathways
Zhang et al. (2020), *Am J Clin Pathol *153	FCM PB screening for hematologic malignancy	ML tool with clinical information & laboratory values as input data	Decision tree model for triaging PB FCM specimen; not considered appropriate for screening of a general population
Flores-Montero et al. (2019), *J Immunol Methods*	Automated identification of PB lymphocyte subsets for chronic lymphoproliferative disorders	EuroFlow Lymphoid Screening Tube (LST) data base	Reliable & reproducible tool for fast identification of normal vs pathological B and T/NK lymphocytes
*Lymphoid neoplasms*			
Scheuermann et al. (2017), *Clin Lab Med* 37	FCM based identification of diagnostic cell populations in CLL patient samples	FLOCK-(Flow clustering without K) based computational pipeline (publically available for open use, http://www.immport.org)	Clinical validation of computational approaches for use in the clinical laboratory
Moraes et al. (2019), *Comput Methods Programs Biomed* 178	FCM-based, automated classification of mature lymphoid neoplasms	Decision-tree approach for the differential diagnosis using logistic function nodes	Validated scheme in diagnostic samples
Gaidano et al. (2020), *Cancers* 12	FCM-based classification of mature lymphoid neoplasms	ML based on manual FCM analysis of clinical cases from a database	High accuracy for common clinicopathological entities
Salama et al. (2022), Cancers 14	FCM MRD in CLL	Deep neuronal network for MRD detection; “human-in-the-loop” AI approach	High accuracy in CLL MRD detection; provides framework for testing in other hematologic disorders
Simonson et al. (2021), *Am J Clin Pathol *156	FCM ML in classic Hodgkin Lymphoma	CNN for detecting cHL using FCM data (two-dimensional histograms)	New ML algorithm with focus on explainability & visualization (Shapley additive explanation value)
Nanaa et al. (2021), *Pathology* 53	Literature review; AI application in the diagnostics of leukemia & lymphoma	AI algorithms applied to digital morphology and FCM	High accuracy of AI tools in diagnostic hematopathology
Zhao et al. (2020), *Cytometry Part A*, 97A	FCM classification of mature B cell neoplasms	Transformation of FCM raw data into a single image file (SOM), further analyzed by CNN for pattern recognition	SOM-CNN-based classification method able to differentiate eight B‑NHL subtypes & normal controls with high accuracy
Nguyen et al. (2023), Br *J Hematol* 00	FCM CLL MRD	Flow SOM	Feasibility & value of automated FCM analysis in the clinical laboratory
*Plasma cell disorders*			
Sanoja-Flores et al. (2018), *Blood Cancer J* 8	Characterization of MGUS & PCM	Next-generation FCM approach on circulating plasma cells	Correlation with diagnostic and prognostic disease categories
Clichet et al. (2022), *Br J Hematol *196	FCM classification of plasma cell dyscrasias (MGUS, SPCM, PCM)	Immunophenotypic profile analysis (FCM) based on a gradient boosting machine (GBM) algorithm using seven FCM parameters	Expression of CD27 & CD38 was found crucial to discriminate MGUS from MM
Flores-Montero et al. (2017), *Leukemia* 31	MRD plasma cell myeloma	EuroFlow-based NGS FCM; standardized approach for MRD detection	Improved sensitivity for MRD detection; prognostic value; ready for implementation in routine diagnostics
*Myelodysplastic syndrome*			
Barreau et al. (2019), *Cytom B Clin Cytom* 98	Evaluation of maturation of granulocytes & monocytes	Manual expert analyzed FCM score	Improvement of accuracy of FCM diagnosis
Duetz et al. (2021), *Cytometry* 99	Computational workflow for MDS diagnosis; distinction between MDS and non-neoplastic cytopenias	FlowSOM, random forest (ML qualifier)	Workflow outperformed the conventional, expert analyzed FCM scores with respect to accuracy, objectivity, and turn-around time
Clichet et al. (2023), *Haematologica*, online, *ahead of print*	FCM based model to predict MDS	ML model based on FCM parameters selected by Boruta algorithm	Improved the sensitivity of the Ogata score; used both in low & high risk MDS
Porwit et al. (2022), *Cytometry B Clin Cytom *102	FCM analysis of normal BM & BM from MDS patients targeting erythropoiesis	FlowSOM Identification of 6 subpopulations of erythropoietic precursors in normal BM & additional 18 subsets in MDS	Unsupervised clustering analysis of FCM data disclosed subtle alterations not detectable by FCM supervised analysis
*Acute leukemia and MRD*			
Monaghan et al. (2022*), Am J Clin Pathol *157	Assessment of BM in unclear cytopenia and/or AL	ML model based on a 37-parameter FCM panel for AL diagnosis & classification	Use of three parameters including light scatter properties demonstrated excellent performance
Zhong et al. (2022), *Diagnostics* 12	FCM AML diagnostics	Automated gating & AML classification based on multiple ML-based techniques	Rapid and effective technique; integration of other test findings
Vial et al. (2021), *Cancers* 13	MRD in AML	Combined unsupervised FlowSOM & Kaluza software	Powerful tool for MRD, particularly applicable to AML without molecular markers
Porwit & Bene (2021), *Hematology* 2	Plasmacytoid dendritic cell compartment in AL with/without *RUNX1* mutation	Unsupervised FCM analysis & clustering	High interpatient variability disclosed by unsupervised analysis
Reiter et al. (2019), *Cytometry* Part A 95A	ALL MRD analysis	Multiple Gaussian mixture models (GMM) for automated MRD assessment	Objective & standardized tool for possible use across different laboratories
Ko et al. (2018), *EBioMedicine* 37	MRD in AML and MDS	FCM algorithm for MRD detection (Gaussian mixture model)	High accuracy with short turn-around time; high prognostic significance; ability to integrate with other clinical tests
Lhermitte et al. (2018), *Leukemia* 32	FCM-based diagnosis & classification of AL	Database-guided analysis used for standardized interpretation of the EuroFlow AL orientation tube	Accurate selection of relevant panels for different AL types; computer-supported reproducible classification even without using the full panels

*BM* bone marrow, *PB* peripheral blood, *CNN* convolutional neural networks, *FlowSOM* self-organizing map, *AI* artificial intelligence, *ML* machine learning, *FCM* flow cytometry, *MRD* measurable residual disease, *CLL* chronic lymphatic leukemia, *MF* marrow fibrosis, *AA* aplastic anemia, *MDS* myelodysplastic neoplasm, *MPN* myeloproliferative neoplasm, *MGUS* monoclonal gammopathy of undetermined significance, *(S)PCM* (smouldering) plasma cell myeloma, *NGS* next generation sequencing, *ALL* acute lymphoblastic leukemia, *AL* acute leukemia, *AML* acute myeloid leukemia

FlowSOM, Bioconductor (10.81129/B9.bioc.FlowSOM)

## Quick evaluation of unclear cytopenia

**Fig. 1:**
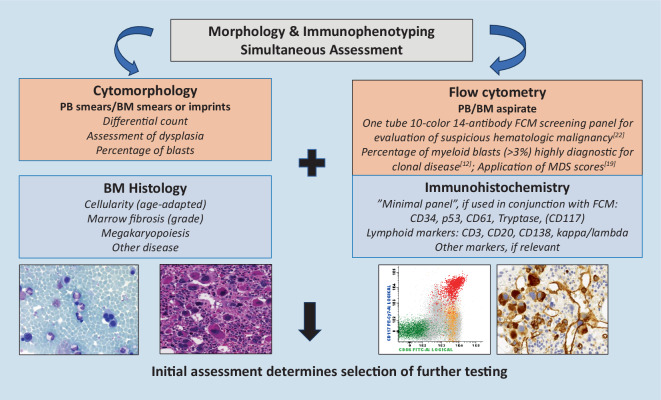
Diagnostic approach to unclear cytopenia and suspicious hematologic malignancy

Unclear cytopenia is the most common indication for BM examination and can have numerous causes, both secondary (reactive) and neoplastic. Chronic (autoimmune) disease, infection, nutritional deficiencies, and treatment-related cytopenia are common non-neoplastic causes. Among hematopoietic neoplasms, the most common causes of new onset cytopenia are MDS and acute myeloid leukemias (AML) in adults and acute lymphoblastic leukemia (ALL) in children. Mature B cell neoplasms can also lead to pancytopenia, but such presentations, apart from hairy cell leukemia, are rare unless in cases with significant BM infiltration. Anemia is a presenting hallmark of plasma cell myeloma, and concomitant thrombocytopenia and granulocytopenia can be seen in up to 10% of patients at the time of diagnosis. In these situations, FCIP is extremely helpful to either confirm or to rule out a hematological neoplasm, particularly in the acute clinical setting of unclear (pan)cytopenia and in patients presenting with cytopenia and secondary dysplasia, mimicking MDS. This basic diagnostic approach is illustrated in Fig. 1 and by the following three clinical cases:

### Case 1

**Fig. 2:**
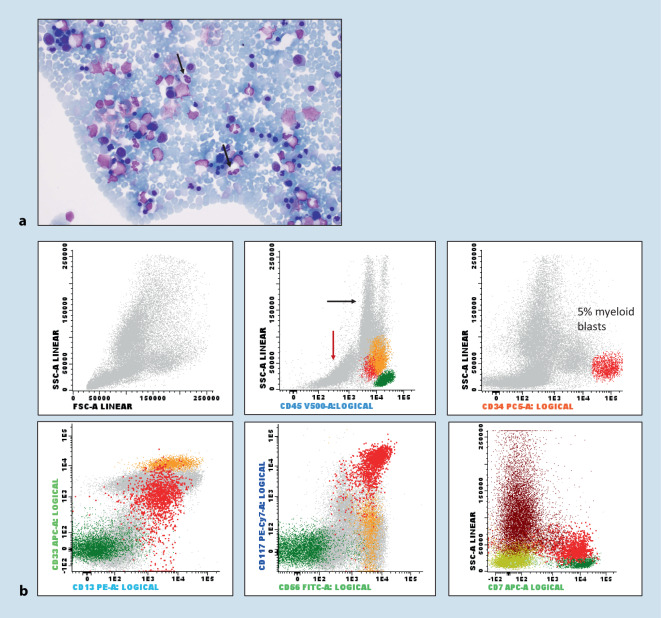
Case 1. **a **Bone marrow aspirate smears show marked dyserythropoiesis (bi-/multinuclearity, dysmorphic nuclei) and left-shifted granulopoiesis with dysplastic, hypogranulated neutrophils (*black arrows*). **b **Flow cytometric analysis of the bone marrow aspirate shows an expanded erythropoiesis (*red arrow*), decreased side scatter (*black arrow*), and 5% myeloid blasts with bright CD34 expression and aberrant expression of CD56 and CD7

A 4-year-old child with unclear anemia and thrombocytopenia. BM smears showed marked erythroid dysplasia and 5% blasts with aberrant myeloid immunophenotype by FCM (Fig. 2). The combined assessment of BM cytomorphology and FCM supported the diagnosis of a myeloid neoplasm (short turn-over time < 2 h) despite the young age of the patient, and provided guidance for subsequent cytogenetic (*here*: normal karyotype) and molecular testing (germline *RUNX1* mutation).

### Case 2

**Fig. 3:**
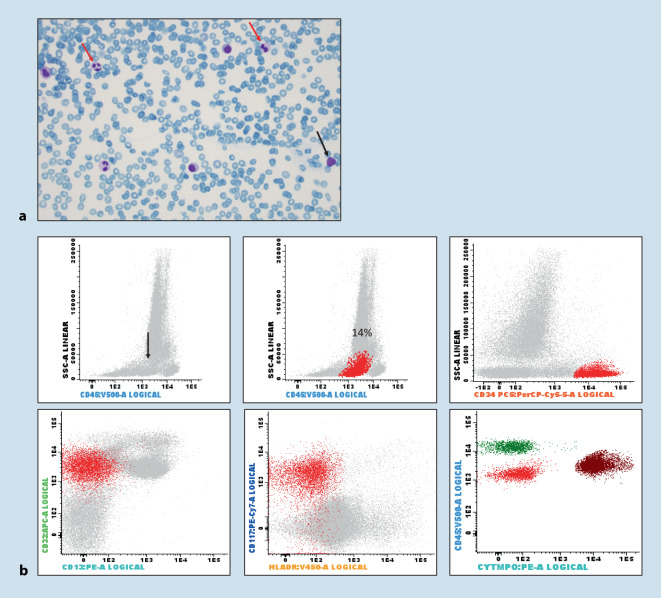
Case 2. **a **Bone marrow aspirate smears are hemodiluted with presence of dysplastic neutrophils (*red arrow*) and occasional blasts (*black arrow*). **b** Flow cytometric analysis of the bone marrow aspirate shows an expanded CD34+ population in the CD45dim blast gate (*black arrow*) with aberrant myeloid immunophenotype (CD13-, HLADR-, MPO-)

A 79-year-old woman with unclear pancytopenia. BM smears were severely hemodiluted with presence of dysplastic neutrophils, but no apparent blast increase. However, FCM of the BM aspirate showed an expanded population of myeloid progenitors (> 10% of total BM cells) with aberrant immunophenotype (Fig. 3). Despite poor quality hemodiluted BM smears, a quick (preliminary) diagnosis of a myeloid neoplasm, suspicious for MDS (with excess blasts) was made, supported by FCM findings. The diagnosis was then confirmed by BM histology; additional studies showed MDS-related cytogenetic aberrancies.

### Case 3

**Fig. 4:**
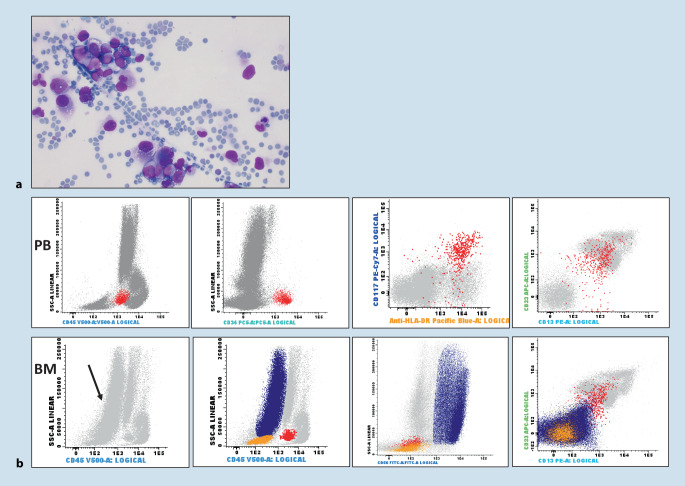
Case 3. **a **Bone marrow (*BM*) aspirate smears show an increase of atypical, blast-like cells with vacuolated cytoplasm. **b **Flow cytometric analysis of the peripheral blood (*PB*) shows a small population of myeloid progenitors with normal immunophenotype (1.7% of leukocytes). Analysis of the BM aspirate displays an abnormal, CD56+/CD45-cellpopulation (56% of all cells; *in blue*), negative for all other myeloid/lymphoid in an expanded lymphoid/myeloid panel (*not shown*); CD45-erythroid progenitors (*in orange*) and normal myeloid progenitor cells (*in red*) represented 5 and 0.6% of all BM cells, respectively

An 18-year-old man with unclear pancytopenia. The PB smear showed mature leukocytes and 2% myeloid blasts without apparent immunophenotypic alterations (Fig. 4). The presence of normal myeloid blasts was reported, leaving the possible cause open. Subsequent BM aspirate smears showed an increased number of atypical, blast-like cells, positive for CD56 but negative for CD45 and all myeloid/lymphoid markers by FCM, which helped to rapidly exclude a hematological malignancy. The final diagnosis of a secondary spread of a rhabdomyosarcoma was established on the BM trephine, supported by immunohistochemistry.

These three clinical examples illustrate that FCM, as an auxiliary tool to cytomorphology, is extremely helpful in acute situations of unknown cytopenias, particularly if smears are inconclusive or of poor quality, or, as in the first case, when samples are from younger individuals or children with dysplastic findings but without obvious blast increase. A previously described practical immunophenotyping strategy with 10-color FCM panels enables the comprehensive evaluation of patients with unclear cytopenia(s) and has been adapted in our laboratory for routine use [22].

## Myelodysplastic neoplasms

The myelodysplastic neoplasms are a group of clonal hematopoietic stem cell diseases characterized by cytopenia, morphologic dysplasia, ineffective hematopoiesis, recurrent cytogenetic abnormalities in ≈50%, and somatic mutations in ≈90% of patients [1, 14]. The current gold standard and first step in the initial diagnostic work-up of unclear cytopenia/suspicious MDS, after the exclusion of other causes of cytopenia, is the cytomorphological examination of the peripheral blood (PB) and bone marrow (BM). Importantly, dysplasia is not specific for MDS and may be present along with other disease in the BM, drug-related, or secondary to other non-neoplastic conditions. The diagnosis of lower-risk MDS subtypes (without excess blasts) can be particularly challenging on morphologic grounds. Importantly, FCM can detect even subtle immunophenotypic aberrancies in the precursor and maturing erythroid, myeloid, and monocytic populations in BM aspirates.

Numerous publications have addressed immunophenotypic abnormalities in MDS patients over the past two to three decades. Most of the earlier studies were qualitative and correlative in nature, identifying FCM aberrancies that correspond to the diagnosis of MDS. Since about the 2010s, there has been a stronger advocacy within the FCM community for a quantitative approach and standardization among different laboratories. A series of consensus guidelines and multicenter studies have been published by the European Leukemia Net (ELN) MDS working group on the use of FCM in the diagnostic work-up of MDS, which were recently updated in a special issue of *Clinical Cytometry*, including pre-analytical, analytical, and technical considerations as well as research results from the members of the group [12, 21, 28, 29]. A multicenter prospective evaluation of FCM aberrancies by the ELNiMDS Flow working group led to the recognition of 17 immunophenotypic markers that were independently related to MDS. FCM showed 80% concordance with cytomorphology when applying a lower cut-off of three aberrant markers [12]. Importantly, this was independent of the number of affected cell lineages, in contrast to previously established guidelines and recommendations. However, there is no single specific FCM marker for MDS and the megakaryocytic lineage is best assessed by cytomorphology in combination with BM histology.

FCIP can help with blast enumeration and identify phenotypically aberrant blasts and be used for treatment follow-up by monitoring previously detected immunophenotypic abnormalities. The percentage of myeloid progenitor cells is informative, but should be correlated to the blast count by routine cytomorphology and BM histology including CD34 immunohistochemistry [25]. Aberrant immunophenotypes of CD34^+^ cells may indicate dysplasia per se and the finding of > 2% CD34^+^ myeloid progenitors was highly associated with MDS [12].

Rarely, MDS may be associated with clonal or non-clonal proliferations of NK and T‑NK cells or large granular lymphocytes in the PB. These populations can be detected and quantified by an appropriate T‑cell FCM panel, including *TRBC1* for the assessment of T cell clonality. FCM screening for the detection of NK or T‑NK cells is a frequently asked assay in neutropenic patients. Simultaneous cytomorphologic examination is highly recommended and the detection of dysplastic neutrophils, particularly in patients with additional anemia and/or thrombocytopenia, should prompt BM examination to rule out MDS.

Several diagnostic MDS FCM scores have been published (recently reviewed in [19]), including the FCM scoring system, the Ogata score, the RED score and ELN-NEC, and the integrated flow score (iFS), the latter encompassing the analysis of myeloid progenitors, granulo- and monopoiesis, and nucleated erythroid cells. The most commonly used MDS FCM score is the Ogata score, based on four parameters [20]. Most MDS-FCM scores have been validated in clinical studies, comparing MDS patients to patients with secondary (reactive) cytopenias and normal controls. The iFS was shown to have the highest accuracy with respect to MDS diagnosis. [19].

## Myeloproliferative neoplasms

The role of FCIP in the diagnosis and classification of myeloproliferative neoplasms (MPN) is not well defined. The integration of molecular findings with BM morphology and PB counts remains the cornerstone of diagnosis, including the *BCR-ABL1* translocation in chronic myeloid leukemia (CML), the MPN-associated mutations *JAK2 V617F, JAK2* exon 12, *MPL 515L/K*, and calreticulin (*CALR*) for the classical *BCR::ABL1*-negative MPN subtypes, and the presence of driver mutations in the colony-stimulating factor 3 receptor (*CSF3R*) in chronic neutrophilic leukemia. However, MPNs exhibit frequent immunophenotypic alterations in both myeloid progenitors and in the maturing myelomonocytic compartment (summarized in Table 1, including references from a literature review). Published data demonstrate that these changes are part of constellational findings in MPN and correlated with adverse clinical and morphological features, such as, for example, an increased blast percentage and an abnormal karyotype. Integration of the Ogata score using FCM analysis of the BM aspirate in patients with PMF resulted in improved prognostic stratification. The enumeration and immunophenotypic characterization of blasts by FCM remains important for the distinction between reactive versus neoplastic conditions and between an accelerated phase of MPN (MPN-AP) and de novo acute leukemia (AL). Importantly, patients with lower blast percentages (5–9%) may have a similar clinical course and prognosis to patients with MPN-AP, and FCIP can help in the early detection of progressive disease. Herborg et al. found that enumeration of circulating immature cells by FCM, including aberrant surface expression, was a promising discriminative tool in MPN diagnostics and a means of monitoring patients longitudinally [8].

In the ICC, immunophenotyping is now included as a criterium for the detection of lymphoblastic crisis in chronic myeloid leukemia (CML), using a threshold of 5% lymphoblasts in the PB or BM [1]. However, this cut-off is arbitrary, and some data suggest that the finding of any bona fide lymphoblasts should raise concern that a lymphoblastic crisis may be imminent. For this reason, a specific cut-off is not included in the WHO-HAEM5, while the presence of “increased” lymphoblasts still remains one of the criteria for blast phase in CML. Since FCM can identify aberrant lymphoid blast populations at much lower levels (< 0.1%), integration in the routine follow-up of CML patients using similar panels as for ALL MRD measurement should be considered.

FCIP can also assist in the diagnostic work-up of other subtypes within the MPN category, particularly in cases with a CMML-like clinical presentation. For example, the clinical and morphologic findings in CEL, NOS, may overlap those of other MPNs, MDS, and MDS/MPN, as illustrated by a previous BM workshop report [11]. In these situations, FCIP can provide hints for correct classification through the identification of MDS-related abnormalities and/or the analysis of monocytic subpopulations, as discussed in the following section. Recent studies recognize the potential value of proliferative and apoptotic indices as diagnostic and prognostic markers in MPN [18].

## Myelodyplastic/myeloproliferative neoplasms

This category of myeloid neoplasms is defined by overlapping pathologic and molecular features of MDS and MPN, clinically manifesting with various combinations of cytopenia and cytoses. The prototype and most common MDS/MPN is chronic myelomonocytic leukemia. Abnormal partitioning of PB monocyte subsets has been introduced as a new supporting diagnostic criterion, based on the observation that an increase of “classical” monocytes (CD14^++^, CD16^−^) was highly sensitive and specific for CMML [26]. Subsequent studies have validated these findings and confirmed the ability to distinguish CMML from both reactive monocytosis and other myeloid neoplasms presenting with monocytosis (Table 1, including references from a literature review). FCIP may also assist to separate the two CMML subtypes, myelodysplastic vs. myeloproliferative, at an immunophenotypic level by using MDS-adapted FCM panels according to ELN recommendations.

The ICC recognizes clonal monocytosis of undetermined significance (CMUS) as a CMML precursor condition, based on persistent monocytosis in the presence of a myeloid neoplasm-associated mutation(s), but without BM morphologic findings of CMML. While FCIP of the PB is not listed as a co-criterion, it could potentially be used as a screening test and predictive marker for the presence of a somatic mutation and for developing CMML.

Other flow cytometric alterations in CMML (see literature review in Table 1 for additional references) are well described and like those found in MDS. For example, expression of CD56 is highly sensitive and specific for a diagnosis of CMML, but only when combined with other immunophenotypic features, including reduced expression of myeloid antigens and ≥ 20% immature monocytes, since CD56 overexpression can also be seen in non-neoplastic conditions.

Outside of CMML, few larger studies have evaluated FCM findings in MDS/MPN overlap. Li et al. found that all studied patients with MDS/MPN demonstrated at least one abnormality by FCM; myeloblast abnormalities were the most common phenotypic aberrancy detected [16]. Although the findings were not entity specific, their presence was helpful in substantiating a diagnosis of a myeloid neoplasm and helping to exclude a reactive process. Finally, FCM can also be used as a predictive marker for treatment response in this group of patients.

## Myeloid/lymphoid neoplasms with eosinophilia and tyrosine kinase gene fusions

The myeloid/lymphoid neoplasms with eosinophilia and TK gene fusions (M/LN-eo-TK) represent a rare and challenging group of hematological neoplasms with highly variable clinical and morphologic presentation and course of disease [1, 14]. M/LN-eo-TK frequently manifest as a chronic myeloid neoplasm with or without eosinophilia resembling CEL, NOS, other MPN, MDS/MPN, or MDS. Other presentations include T or B acute lymphoblastic leukemia/lymphoma (ALL), AML, the blast phase of MPN, or mixed-phenotype acute leukemia (MPAL). Importantly, cases may present with disparate stages of disease (chronic vs. acute or transformed), disparate cell lineages, as primary extramedullary (nodal/extranodal) disease, or manifest first during follow-up and/or after treatment of the original diagnosis. Abnormal mast cell proliferations, detected by FCM or immunohistochemistry (IHC), are frequent findings in M/LN-eo with any of the recurrent TK fusion genes. Taken together, FCIP plays an important and central role in the diagnostic work-up of M/LN-eo for the detection of aberrant (sub)populations or multilineage involvement, as illustrated by numerous single case reports, including children, multicenter studies, and recent BM workshop reports [23, 24].

## Mastocytosis

Mastocytosis is characterized by neoplastic proliferation of abnormal mast cells in at least one organ system, including the skin and BM, and can be divided into cutaneous mastocytosis (CM), systemic mastocytosis (SM), and mast cell sarcoma (MCS) [1, 14]. Advanced SM compromises aggressive SM, SM with an associated myeloid neoplasm (SM-AMN), and mast cell leukemia (MCL). In both classifications (ICC/WHO-HAEM5), the major diagnostic criteria for SM remain largely based on morphology, supported by immunophenotypic analysis. FCIP can be used to identify mast cell populations, which are recognized by their unique phenotype (CD117^++^, CD45^+^, CD38^−^, CD33^+^, CD13^±^, CD11c^+^, CD11b^±^, CD71^+^). Neoplastic mast cells usually demonstrate aberrant expression of CD25 and CD2, and may show an altered intensity of antigens which are normally expressed on mast cells. The recognition of an abnormal mast cell immunophenotype is one of the minor diagnostic criteria for SM in both classifications. CD30 expression, either by FCM or IHC, can be detected in up to 80–90% in SM and is now accepted as an additional marker in defining immunophenotypic aberrancy of MCs.

Normally, mast cells account for < 0.1% of total BM cells, and are usually < 2% of cells even in SM. Therefore, FCM analysis for mast cells require strategies similar to those used for the detection of measurable residual disease (MRD). FCIP provides a more sensitive method as compared to IHC for detecting abnormal mast cells and can be useful for the identification of circulating mast cells, present in nearly half of the patients with indolent SM and almost all patients with advanced SM [7]. Finally, FCM can also be helpful for the detection of an associated myeloid neoplasm (SM-AMN) or a concurrent lymphoid or plasma cell neoplasm.

## Acute myeloid leukemia

Acute myeloid leukemia (AML) classification relies mainly on cytogenetic and molecular data [1, 14]. However, the overall AML classification structure continues to emphasize the integration of clinical, morphologic, immunophenotypic, and genomic findings. In AML, FCM remains an essential tool for rapid diagnosis and lineage assignment. Within the categories of AML not otherwise specified, NOS, (ICC)/AML with differentiation, FCM is necessary to confirm AML with minimal differentiation, to detect monocytic and megakaryoblastic differentiation and to diagnose mixed-phenotype acute leukemias (MPAL). Characteristic immunophenotypic findings in AML with main specific genetic abnormalities are well known and have been described and reviewed in previous classifications [27] and recent publications [17]. Importantly, FCM analysis, together with cytomorphology, is essential for prompt diagnosis and treatment of acute promyelocytic leukemia (APL). The combination of cytomorphology and FCIP permits the diagnosis with a high degree of certainty; however, distinction from APL mimics (e.g., *NPM1*-mutated, other AML with monocytic differentiation, *KMT2A*-rearranged AML) can be challenging. Several gating strategies have been explored to distinguish APL from AML subtypes with APL-like immunophenotypes, including the use of radar plots [6]. More recently, Fang et al*.* (2022) compared patients with APL to *NPM1*-mutated AML and suggested that CD2 and/or CD34 expression, along with uniform CD13 and CD64 positivity, is more consistent with microgranular APL [5].

AML with *RUNX1* mutation has been associated with mixed-phenotype acute leukemia (MPAL) and with expansion of the plasmacytoid dendritic cell compartment. AML with myelodysplasia-related genetic abnormalities often carry immunophenotypic features like those described in MDS. However, the simultaneous occurrence of mutations and cytogenetic alterations makes it difficult to identify specific patterns associated with certain mutations. FCIP is also needed for the diagnosis of MPAL; however, criteria for lineage assignment are still a matter of debate and many cases previously assigned to this category with predominant myeloid blast populations will now be diagnosed as AML based on cytogenetic or mutation findings [3].

Finally, FCM plays an important role in MRD testing, which is a strong predictor of relapse and shorter survival in AML patients [9]. Nevertheless, MRD measurements are not yet used routinely to guide therapeutic decisions in AML, partly due to the lack of uniformity in methodologies used for MRD detection and interpretation. The ELN MRD working group has published guidelines for MRD analysis, including the harmonized use of an integrated diagnostic “leukemia-associated immunophenotype” (LAIP) and “different from normal” (DfN) approach with a set of MRD core markers [9]. Since relapses also occur in MRD-negative patients, further research is focused on measuring the frequency of residual leukemic stem cells, which may be resistant to therapy [13].

Apart from the role of FCM as a diagnostic and prognostic tool, it is also of value in patients being considered for targeted therapy and treatment with chimeric antigen receptor T cells (CAR-T) by demonstrating the expression of cell surface antigens (e.g., CD33, CD123) on leukemic blasts [10].

## Artificial intelligence and machine learning

The application of AI strategies and ML for the analysis of complex FCM data, recently reviewed in [2, 15, 30], has opened a new, rapidly expanding era. Efforts to develop, validate, and disseminate automated computational methods for FCM data analysis can help overcome the limitations of manual analysis and provide efficient and data-driven diagnostic applications. An increasing number of mostly recent studies underline the applicability of AI tools for the analysis of FCM data in hematological malignancies (Table 2). Similarly, modern cyto-and histomorphology is evolving towards AI-assisted “digital microscopy,” allowing automated pattern recognition and classification of acquired cell images. A more detailed review of these studies is beyond the scope of this article. Briefly, ML models have demonstrated human-level performance using FCM data for the classification of chronic lymphoproliferative diseases and for the diagnosis, classification, and MRD testing of acute leukemias. AI applications in MDS include the use of convolutional neural networks to assess dysplasia, the development of an AI-assisted prediction score for MDS diagnosis [4], and models that provide a link between morphology, mutational status, and prognosis. Finally, markers for biological behavior, such as proliferative and apoptotic parameters, have been proposed as interesting candidates for incorporation into such automated approaches [18]. Data from the literature provide evidence that AI-driven FCM diagnostics allow reliable and comprehensive (multidimensional) automated analysis of large immunophenotypic datasets, and clinical applications appear time efficient and potentially harmonizable across laboratories.

## Concluding remarks

Flow cytometry is an indispensable tool for quick diagnosis, classification, and follow-up of patients with myeloid neoplasms, particularly AML and MDS. Immunophenotypic alterations are already used as diagnostic criteria in several disease entities in the WHO/ICC classifications. FCIP should therefore be included into routine diagnostic algorithms in clinical hematology, supported by artificial intelligence approaches. Clinical validation of these computational approaches is ongoing and essential to realize their true potential for use in the clinical diagnostic laboratory.

### Key points.

- Flow cytometry is a valuable tool for rapid diagnosis, classification, prognosis, and monitoring of hematologic neoplasms.
- Immunophenotypic profiles can identify underlying genomic alterations and be useful to highlight prognostically relevant differences within subgroups of acute myeloid leukemia.
- Further standardization and harmonization will become essential for implementing clinical flow cytometry in routine diagnostic evaluations in chronic myeloid neoplasms.
- Artificial intelligence and machine learning offer an effective way to elaborate and interpret large-scale datasets and help to refine diagnostics.

## Declarations

All investigations on humans or human tissues described in this article were carried out with the approval of the responsible ethics committee, in accordance with national law and the Declaration of Helsinki from 1975 (in its current revised form). The investigations were carried out according to the specifications of the Central Ethics Committee of the Federal Medical Council.

The supplement containing this article is not sponsored by industry.
